# The PRIDE database at 20 years: 2025 update

**DOI:** 10.1093/nar/gkae1011

**Published:** 2024-11-04

**Authors:** Yasset Perez-Riverol, Chakradhar Bandla, Deepti J Kundu, Selvakumar Kamatchinathan, Jingwen Bai, Suresh Hewapathirana, Nithu Sara John, Ananth Prakash, Mathias Walzer, Shengbo Wang, Juan Antonio Vizcaíno

**Affiliations:** European Molecular Biology Laboratory, European Bioinformatics Institute (EMBL-EBI), Wellcome Trust Genome Campus, Hinxton, Cambridge, CB10 1SD, UK; European Molecular Biology Laboratory, European Bioinformatics Institute (EMBL-EBI), Wellcome Trust Genome Campus, Hinxton, Cambridge, CB10 1SD, UK; European Molecular Biology Laboratory, European Bioinformatics Institute (EMBL-EBI), Wellcome Trust Genome Campus, Hinxton, Cambridge, CB10 1SD, UK; European Molecular Biology Laboratory, European Bioinformatics Institute (EMBL-EBI), Wellcome Trust Genome Campus, Hinxton, Cambridge, CB10 1SD, UK; European Molecular Biology Laboratory, European Bioinformatics Institute (EMBL-EBI), Wellcome Trust Genome Campus, Hinxton, Cambridge, CB10 1SD, UK; European Molecular Biology Laboratory, European Bioinformatics Institute (EMBL-EBI), Wellcome Trust Genome Campus, Hinxton, Cambridge, CB10 1SD, UK; European Molecular Biology Laboratory, European Bioinformatics Institute (EMBL-EBI), Wellcome Trust Genome Campus, Hinxton, Cambridge, CB10 1SD, UK; European Molecular Biology Laboratory, European Bioinformatics Institute (EMBL-EBI), Wellcome Trust Genome Campus, Hinxton, Cambridge, CB10 1SD, UK; European Molecular Biology Laboratory, European Bioinformatics Institute (EMBL-EBI), Wellcome Trust Genome Campus, Hinxton, Cambridge, CB10 1SD, UK; European Molecular Biology Laboratory, European Bioinformatics Institute (EMBL-EBI), Wellcome Trust Genome Campus, Hinxton, Cambridge, CB10 1SD, UK; European Molecular Biology Laboratory, European Bioinformatics Institute (EMBL-EBI), Wellcome Trust Genome Campus, Hinxton, Cambridge, CB10 1SD, UK

## Abstract

The PRoteomics IDEntifications (PRIDE) database (https://www.ebi.ac.uk/pride/) is the world’s leading mass spectrometry (MS)-based proteomics data repository and one of the founding members of the ProteomeXchange consortium. This manuscript summarizes the developments in PRIDE resources and related tools for the last three years. The number of submitted datasets to PRIDE Archive (the archival component of PRIDE) has reached on average around 534 datasets per month. This has been possible thanks to continuous improvements in infrastructure such as a new file transfer protocol for very large datasets (Globus), a new data resubmission pipeline and an automatic dataset validation process. Additionally, we will highlight novel activities such as the availability of the PRIDE chatbot (based on the use of open-source Large Language Models), and our work to improve support for MS crosslinking datasets. Furthermore, we will describe how we have increased our efforts to reuse, reanalyze and disseminate high-quality proteomics data into added-value resources such as UniProt, Ensembl and Expression Atlas.

## Graphical Abstract

**Graphical Abstract:**
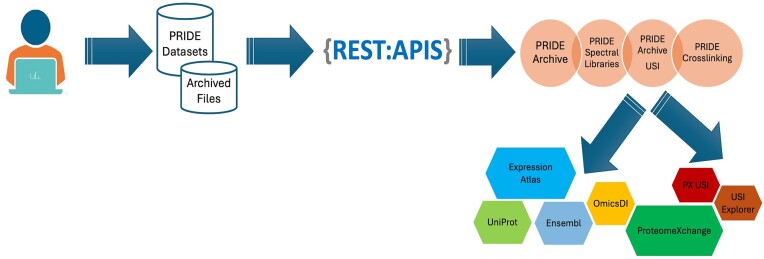

## Introduction

Data sharing in the public domain has become the standard behavior for proteomics researchers and many scientific journals and funding agencies currently mandate open science practices, involving for instance the submission of proteomics datasets to public repositories (1,2). The PRoteomics IDEntifications (PRIDE) database (https://www.ebi.ac.uk/pride/) at the European Bioinformatics Institute (EMBL-EBI, Hinxton, Cambridge, UK) enables public data deposition of mass spectrometry (MS)-based proteomics data, providing access to the experimental data described in scientific publications (3). Started in 2004 (4), PRIDE Archive (the archival component of PRIDE) is the largest data repository for proteomics data worldwide (3,5).

PRIDE stores datasets coming from all MS-based proteomics experimental approaches, including quantitative data-dependent acquisition (DDA) and data-independent acquisition (DIA) bottom-up proteomics, but also, to a smaller extent, datasets generated from other workflows such as e.g. top-down, peptidomics (e.g. immunopeptidomics approaches) or crosslinking proteomics, in parallel with the trends in the field. PRIDE is one of the founders of the ProteomeXchange consortium (https://www.proteomexchange.org) (5,6) bringing together MS-based proteomics data resources worldwide. ProteomeXchange, formally established in 2012, provides globally coordinated standard data submission and dissemination pipelines for proteomics datasets, and promotes open data policies in the field. The resources PeptideAtlas (7), including its related resource PASSEL (PeptideAtlas SRM Experiment Library) (8), MassIVE (9), jPOST (10), iProX (11) and Panorama Public (12), are the members of the consortium in addition to PRIDE. ProteomeXchange provides a common accession number for every submitted dataset and a set of services for public data search and retrieval across the resources.

In December 2022, ProteomeXchange resources were recognized in the initial list of Global Core Biodata Resources (https://globalbiodata.org/what-we-do/global-core-biodata-resources/) by the Global Biodata Coalition, with the aim to highlight those essential biological resources for the scientific community. The PRIDE database is also a core data resource of ELIXIR (http://www.elixir-europe.org) (13), recognizing its key role in the life sciences ecosystem in Europe.

PRIDE, together with the other ProteomeXchange resources, supports the FAIR (Findable, Accessible, Interoperable, Reusable) data principles (14). In the context of interoperability, the PRIDE team has (co)led within the PSI (Proteomics Standards Initiative) (15), the development and implementation of several open standard formats such as mzTab (16), mzIdentML (17,18), mzML (19), ProForma version 2.0 (20), the SDRF-Proteomics (Sample and Data Relationship File) format (21) and the Universal Spectrum Identifiers (USIs) (22), among others, to facilitate the storage, processing and visualization of the deposited proteomics data.

PRIDE resources have two main missions: (i) support data deposition of all types of MS-based proteomics data supporting reproducible research and enabling public data reuse, implementing the FAIR data principles; and (ii) to bring proteomics data closer to life scientists by re-using, disseminating and integrating the data in other resources, including EMBL-EBI’s Ensembl (23), UniProt (24) and Expression Atlas (25), among others.

In this manuscript, we summarize the main PRIDE-related developments in the last three years, since the previous *Nucleic Acids Research* (NAR) database update manuscript was published (3). We will discuss PRIDE Archive and related resources first but will also provide updated information about other ongoing activities including the updates in the data reuse context, performed to disseminate and integrate proteomics data in other resources.

## Current status of the PRIDE ecosystem: resources and tools

**Figure 1.**
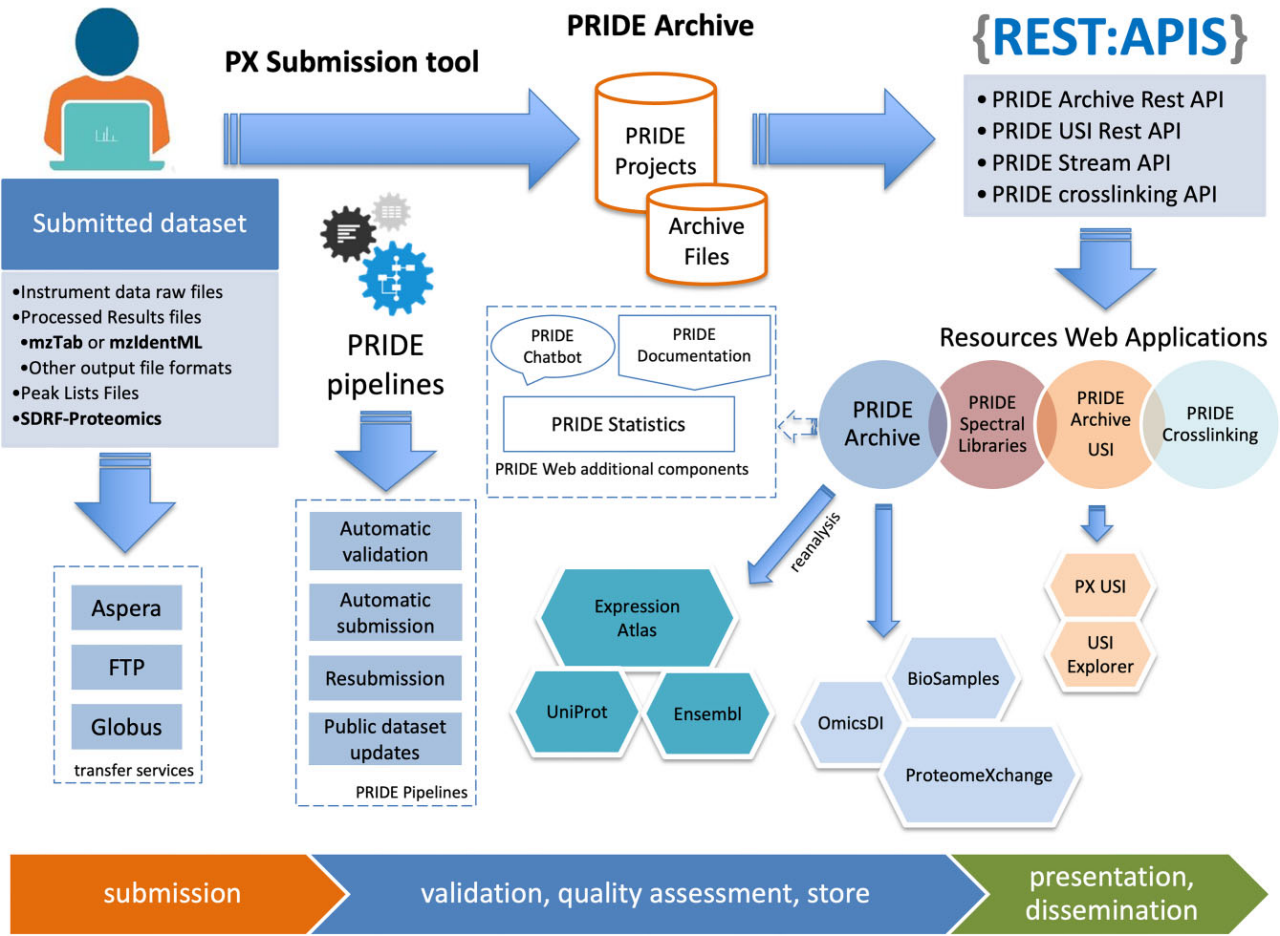
Overview of the PRIDE dataset submission, validation, storage and dissemination process. Researchers submit datasets to PRIDE Archive using the ProteomeXchange Submission Tool, which supports multiple data formats (e.g. MS raw files, processed result files, mzTab, mzIdentML, peak lists and SDRF-Proteomics). Data transfer is facilitated through services like Aspera, FTP, and the newly added Globus service. Submitted datasets are processed by PRIDE Pipelines, which perform automatic validation, submission, resubmission and publication of the private datasets. Validated datasets are stored in the PRIDE Archive, where they can be accessed via various REST APIs (e.g. PRIDE Archive Rest API, PRIDE USI Rest API, PRIDE Stream API and PRIDE Crosslinking API). These datasets are further disseminated through PRIDE’s web applications, including PRIDE Spectral Libraries, PRIDE USI and PRIDE Crosslinking, as well as external resources such as Expression Atlas, UniProt, Ensembl, OmicsDI, BioSamples and ProteomeXchange.

The PRIDE database ecosystem is composed of a set of libraries, desktop tools, databases, large-scale pipelines, Restful APIs (Application Programming Interface) and web applications. Figure 1 illustrates the current PRIDE ecosystem, including web services and data pipelines. Since 2022, the major focus of the PRIDE team in infrastructure-related topics has been put in three major areas: (i) data transfer, including the availability of a new protocol for uploading data to PRIDE Archive, the Globus file transfer service; (ii) Automatic validation and resubmission pipelines which enable to reduce the time it takes for every submitter to get a dataset accession number; and (iii) the development of new services to query and retrieve PRIDE data: PRIDE USI service (https://www.ebi.ac.uk/pride/archive/usi) and the PRIDE Crosslinking resource (https://www.ebi.ac.uk/pride/archive/crosslinking).

A set of open-source Java libraries supported and maintained by the team enables the reading, validation, processing and storage of proteomics data encoded in PSI open file formats (15,26). PRIDE Archive data pipelines (validation, submission, resubmission and publication) make possible the validation and submission of datasets and files in the EMBL-EBI production file system. The team has increased the number of Restful APIs that enable querying the PRIDE data in multiple ways, including searching, retrieving, and streaming private and public datasets, and also to retrieve specific mass spectra using USIs (22).

There are four major web interfaces currently in PRIDE: PRIDE Archive (https://www.ebi.ac.uk/pride/archive), PRIDE Archive USI (https://www.ebi.ac.uk/pride/archive/usi), the PRIDE Crosslinking resource (https://www.ebi.ac.uk/pride/archive/crosslinking) and PRIDE spectral libraries (https://www.ebi.ac.uk/pride/spectrumlibrary). In addition, in Figure 1, it can be observed that PRIDE continues to provide metadata and different proteomics data types to other resources including Expression Atlas (25), Omics Discovery Index (27,28), ProteomeCentral (5) as the common search interface in ProteomeXchange, UniProt (24), Ensembl (23) and BioSamples (29). In the following sections, new resources and improvements in existing services will be described in detail.

### Data submission

PRIDE Archive data submission guidelines, aligned with the ProteomeXchange requirements (3), mandate the inclusion of MS raw files and processed results (peptide/protein identification and quantification). Additional components may include peak list files, protein sequence databases or spectral libraries, scripts and other relevant metadata (30). A tutorial on the submission process is available at the EMBL-EBI online training platform: ‘PRIDE Quick Tour’ (https://www.ebi.ac.uk/training/online/courses/pride-quick-tour/). Data submissions are mostly performed using the stand-alone ProteomeXchange submission tool. The tool enables the provision of the required metadata for each dataset, including title, description and controlled vocabulary/ontology terms including information about species, mass spectrometers or diseases (31), among other pieces of information. The PRIDE data policy explaining how datasets are handled is available at https://www.ebi.ac.uk/pride/markdownpage/datapolicy.

Three major improvements have been implemented to facilitate the data submission process to PRIDE Archive: (i) improvements in the dataset resubmission process, (ii) enabling the data uploads using the Globus data transfer service and (iii) automatic validation and submission of datasets.

#### More granularity in the dataset resubmission process

During the manuscript review process, authors may have to add, modify, or remove files in their submitted datasets. Until recently, making changes to a private submission (i.e. under review) required to perform again a new resubmission of all the files included in the dataset, even if only one file needed to get changed/replaced, leading to unnecessary efforts. This approach was not a major issue when the resubmission process was originally designed (at the time submitted datasets averaged around 10 files, making it feasible to transfer the entire dataset again). However, this methodology has become increasingly impractical in time with the growing average number of samples and raw files per dataset.

**Figure 2.**
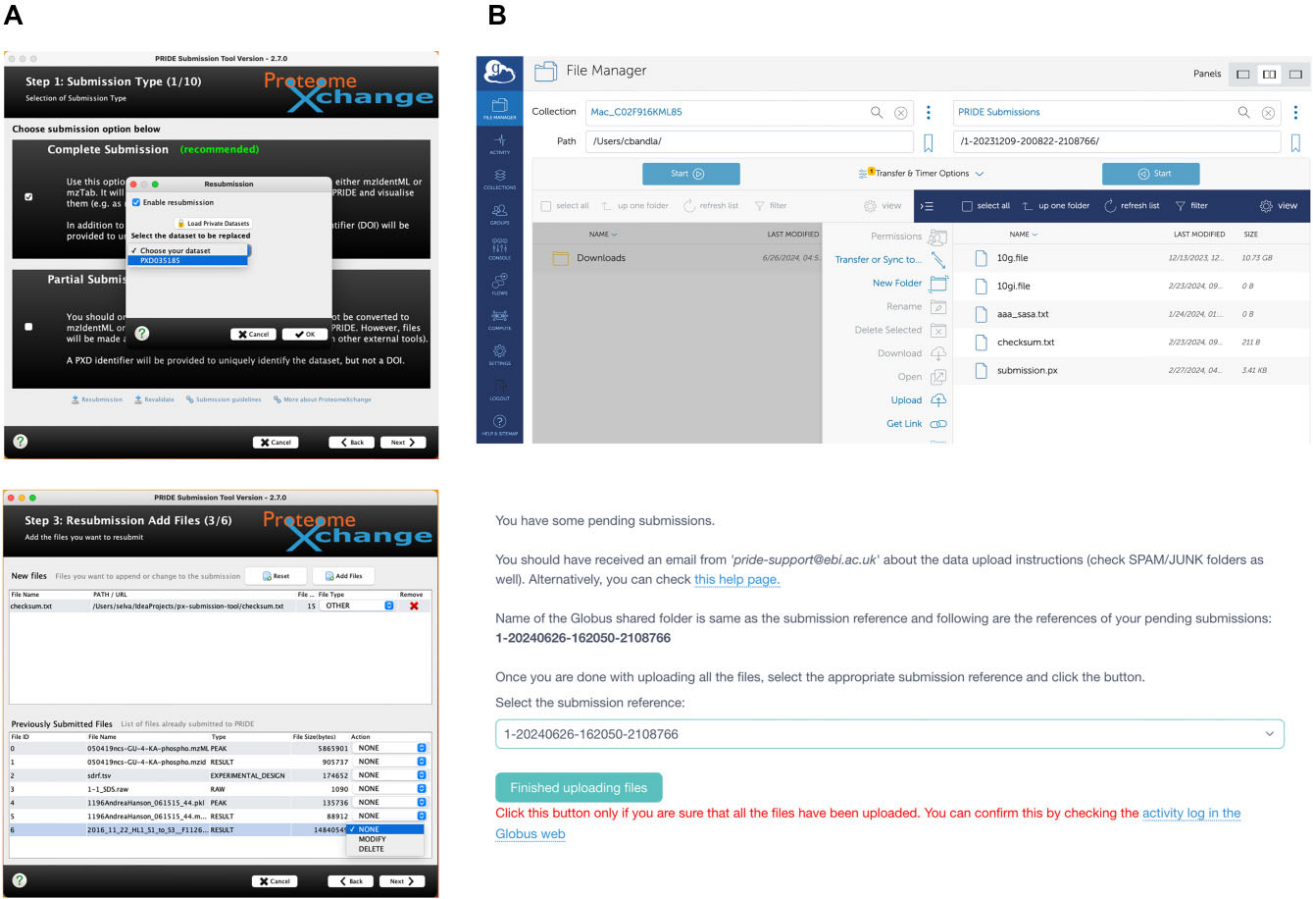
(**A**) ProteomeXchange Submission tool resubmission panels and (**B**) submission using the newly introduced Globus mechanism. The submission tool guides users through a multi-step process for submitting or resubmitting proteomics datasets to PRIDE Archive. (Top left) Step 1 allows users to select from the existing private datasets to perform data resubmissions, and (bottom left) in the next step of the process, the users can upload additional files or replace existing ones, specifying which files are being updated. (Top right) Users can transfer files using Globus, a file transfer service which provides a dedicated folder for each submission. (Bottom right) After uploading all files, users confirm that the data upload process is complete by selecting the appropriate submission reference.

We have implemented a new resubmission system integrated into the ProteomeXchange submission tool (Figure 2A), involving a new dataset resubmission pipeline as well. PRIDE users can now select one of their existing private datasets using the submission tool and choose which files to update, delete, or add. Once the files are uploaded into PRIDE, the resubmission pipeline then validates only the new or modified files, while ensuring the integrity of the entire dataset. Since the release of this feature, it has been extensively used by submitters to modify ‘SEARCH’ files (processed results files from the search engines), which can be often updated during the review process.

#### Globus-based submissions: complementing the FTP and Aspera data transfer protocols

Until recently, data transfers to the PRIDE Archive were performed via FTP (File Transfer Protocol) or Aspera (https://www.ibm.com/products/aspera), with Aspera being the default option due to its faster file transfer speed. However, Aspera is not always accessible at research institutions, since its required ports are often blocked by internal/local IT regulations. Additionally, large datasets can still take several hours to transfer, depending on the users’ internet speed. In such cases, the ProteomeXchange submission tool may freeze, forcing users to restart the submission process.

We have recently introduced a new submission mechanism using the Globus transfer service (https://www.globus.org/data-transfer), offering a third option for performing data submissions alongside the FTP and Aspera protocols. To begin, users should use the ProteomeXchange submission tool to generate the required submission.px file, which contains the submission metadata including also the list of files included in each dataset. The submission.px file, a checksum.txt file (needed to assess the file integrity after the file transfer) and all the files to be submitted, can then be transferred to PRIDE via Globus. Before starting, users must have an account in both PRIDE and Globus; then they must log-into the PRIDE web portal, request a new submission (Figure 2B), and provide their Globus account details. They will receive an email with a folder name for performing the file transfer. After installing and configuring Globus Connect Personal (https://www.globus.org/globus-connect-personal) and following the Globus tutorial, users can select their own created collection and the ‘PRIDE Submissions collection’ in the Globus File Manager. All files, including the checksum.txt and submission.px, should be uploaded to the designated PRIDE folder. Once the upload is complete, users must return to the PRIDE web portal to finalize their submission (https://www.ebi.ac.uk/pride/markdownpage/globus). We recommend using the Globus transfer protocol for large datasets and in institutions where it is not possible to use Aspera due to IT restrictions.

#### Automatic dataset validation and submission

After a dataset is submitted to PRIDE, two steps take place before a submitter receives the dataset accession number: dataset validation and submission. First, in the validation step, the metadata is checked including the controlled vocabularies used, metadata fields (e.g. title), and that the size and integrity of the files submitted are correct (checksum.txt). In the submission step, files are transferred from the staging (submission) area into a more permanent storage system. Metadata is then transferred to a database, enabling submitters to make changes during the manuscript review process without the need to transfer all the data again (see above). Finally, a dataset accession is requested from ProteomeXchange and sent to the submitter.

Until the beginning of 2023, these two processes were manually triggered by a PRIDE curator. While this manual process ensured correctness, it also caused delays in obtaining an accession number due to e.g. increased number of submissions, and/or holiday periods. On average, dataset accessions were issued within 34 hours under this system. We have now introduced a new workflow that uses rules and natural language processing (NLP) pipelines to automate the validation and submission of datasets. This update has reduced the average time to finish data submissions to just 4 minutes.

### Continuing metadata deposition using the SDRF-Proteomics format

Since 2022 PRIDE Archive has supported the submission of general sample metadata and experimental design information using the SDRF-Proteomics format (3,21). This standard tab-delimited format (21) (https://github.com/bigbio/proteomics-metadata-standard) can capture the experimental design and details the relationship between the samples included in a dataset and the corresponding MS data files (raw files).

Submitters can manually add SDRF-Proteomics files to their submitted datasets by selecting ‘EXPERIMENTAL DESIGN’ as the file type in the submission tool. The corresponding experimental design table is accessible through the PRIDE Archive web interface (e.g. dataset PXD047854, https://www.ebi.ac.uk/pride/archive/projects/PXD047854). In the last three years, various tools have enhanced the adoption of SDRF-Proteomics by enabling the annotation, export and reuse of SDRF-Proteomics data from PRIDE. First, lesSDRF (32) is a web-based tool that enables submitters to create templates and annotate their datasets. Additionally, FragPipe (33) enables the export of an SDRF-Proteomics draft file containing search parameters and file names, but not sample details. Furthermore, the quantms workflow (34) facilitates the reuse of public proteomics data using deposited SDRF-Proteomics files. The PRIDE team continues to collaborate with other tool providers (e.g. MaxQuant, Proteome Discoverer) to improve the adoption of SDRF-Proteomics as a standard format for parameter input and experimental design output.

### PRIDE Archive Restful APIs: programmatic access to datasets

The PRIDE RESTful API (https://www.ebi.ac.uk/pride/ws/archive/v2/) enables users to query and access all data within PRIDE resources. The API allows for various queries, such as retrieving datasets by publication date, identifying specific proteins or locating a data file within a given dataset. Its powerful query language supports SQL-based searches by combining multiple keywords (project properties). Additionally, a Python package and tool (https://github.com/PRIDE-Archive/pridepy) have been developed to facilitate programmatic interaction with the PRIDE Archive RESTful API.

Three new APIs have been integrated into PRIDE Archive’s main RESTful web service. The PRIDE file streaming API enables users to transfer files from public datasets using a streaming approach, which processes data in small, manageable chunks rather than loading entire files into memory. More information and a public benchmark comparing FTP, HTTPS and the streaming API can be found in the PRIDE documentation (https://www.ebi.ac.uk/pride/markdownpage/pridefiledownload#benchmarking_data_downloads). The PRIDE Archive USI API (https://www.ebi.ac.uk/pride/molecules/ws/swagger-ui/index.html) allows users to retrieve specific spectra from PRIDE Archive files from Thermo Scientific instruments (see section ‘PRIDEArchive USI: Accessing and Visualizing mass spectra’). Lastly, the PRIDE Crosslinking resource API (https://www.ebi.ac.uk/pride/ws/archive/crosslinking/v2/docs) provides access to data from ‘complete’ crosslinking submissions within the PRIDE Crosslinking resource (see section ‘PRIDE Crosslinking’).

### PRIDE Archive USI: accessing and visualizing mass spectra

Direct access to the identified spectra (or PSMs, Peptide Spectrum Match) within a given dataset enables the evaluation of whether, e.g., novel peptide sequences, post-translational modifications (PTMs) or single amino acid variants (SAAVs) are supported by high-quality, well-annotated mass spectra (2,22). The introduction of USIs has significantly enhanced the transparency of the mass spectral evidence, offering a standardized method for accessing mass spectra data across ProteomeXchange resources. Previously, we developed a first version of the resource, which enabled the retrieval of identified spectra from ‘Complete’ submissions, providing access to over 540 million PSMs at the time. Although the goal was to offer real-time access to all spectra (and not only to those in open formats, as part of ‘Complete’ submissions), this was initially challenging due to PRIDE Archive’s architecture and the difficulty of accessing MS raw data files (from the different MS vendors) from Unix systems.

The current PRIDE Archive USI (https://www.ebi.ac.uk/pride/archive/usi) allows the retrieval of most mass spectra in PRIDE Archive using a USI. Unlike the previous approach of indexing PSMs for ‘Complete’ submissions, the current system reads the provided USI and locates the specified scan directly in the MS raw files. The PRIDE Archive USI APIs leverage the ThermoRawFileParser (35) to extract the scan from the MS raw files, providing access to over 80% of the stored MS raw files (those from Thermo Scientific instruments). Efforts are ongoing to expand access to spectra from other instrument vendors, such as Bruker and SCIEX. Additionally, the PRIDE Archive USI is integrated with the ProteomeCentral API, enabling access to MassIVE, the second-largest resource in ProteomeXchange. Various databases, including MatrisomeDB (36), Scop3P (37) and also ProteomeCentral USI (5), utilize PRIDE Archive USIs to access and visualize PSMs, for peptide identifications included in reanalyzed datasets.

### PRIDE Crosslinking

In the interface between proteomics and structural biology, crosslinking MS is one of the most popular approaches. Due to the increased relevance of structural biology approaches in proteomics, a first version of the PRIDE Crosslinking resource (https://www.ebi.ac.uk/pride/archive/crosslinking) has been developed and recently released, aiming to improve data access and visualization for crosslinking studies and to bridge proteomics and structural biology data. In that context, it provides cross-references to the Protein Data Bank (PDB), including PDBe (PDB in Europe) (38), PDB-Dev and AlphaFoldDB (database of predicted protein structures) (39). The tool xiVIEW (40) has been integrated to enable the visualization of this type of dataset. As of August 2024, PRIDE Crosslinking includes 22 datasets coming from 9 different organisms, encompassing a total of 524 443 peptides coming from 4905 proteins. The number of datasets and overall functionality will grow as new relevant datasets become publicly available and are integrated into the resource (guidelines for submission are available at https://www.ebi.ac.uk/pride/markdownpage/crosslinking). As mentioned above, PRIDE Crosslinking is complemented by an API (https://www.ebi.ac.uk/pride/ws/archive/crosslinking/v2/docs).

### PRIDE chatbot and PRIDE documentation

Artificial intelligence (AI) approaches and Large Language Models (LLMs) are transforming every field where they can be used. We have developed a PRIDE chatbot (https://www.ebi.ac.uk/pride/chatbot/) (41) featuring a web service API, a user-friendly web interface and specialized open-source LLMs. The PRIDE Chatbot has been trained using the PRIDE external documentation. The overall idea is 2-fold: on one hand we aim to help PRIDE users navigate PRIDE documentation, therefore decreasing the time required for the team to reply to user support queries. On the other hand, we would like to improve the dataset search functionality. As of August 2024, two open-source models (Mixtral and llama2-13b-chat) are supported.

During the development of the chatbot, the PRIDE external documentation was optimized by adding new topics and eliminating redundant information. Additionally, the several training videos have been made available, covering the submission process, the SDRF-Proteomics format and the broader ecosystem of PRIDE resources, including tools, web services and the web interface (e.g. https://www.youtube.com/watch?v=VRNumsnYVg0).

### Additional developments

In addition to major advancements in infrastructure, other minor refinements have been implemented. Notably, PRIDE now operates on a complete microservice architecture, where all services—such as databases, file access and search and indexing systems—are provided through microservices (APIs). This architecture enables PRIDE to scale effectively and be deployed in cloud-based Kubernetes environments. The new design allows the PRIDE team to scale each API independently by increasing the number of instances as needed.

Additionally, the ProteomeXchange submission tool now allows submitters to manually select and switch the submission protocol (FTP or Aspera) directly within the interface. In previous versions, users had to modify a configuration file to change the protocol, making the process cumbersome. The current version streamlines this by enabling protocol switching within the interface without requiring users to close the application, edit configuration files or restart the tool.

## PRIDE Archive submission statistics

**Figure 3.**
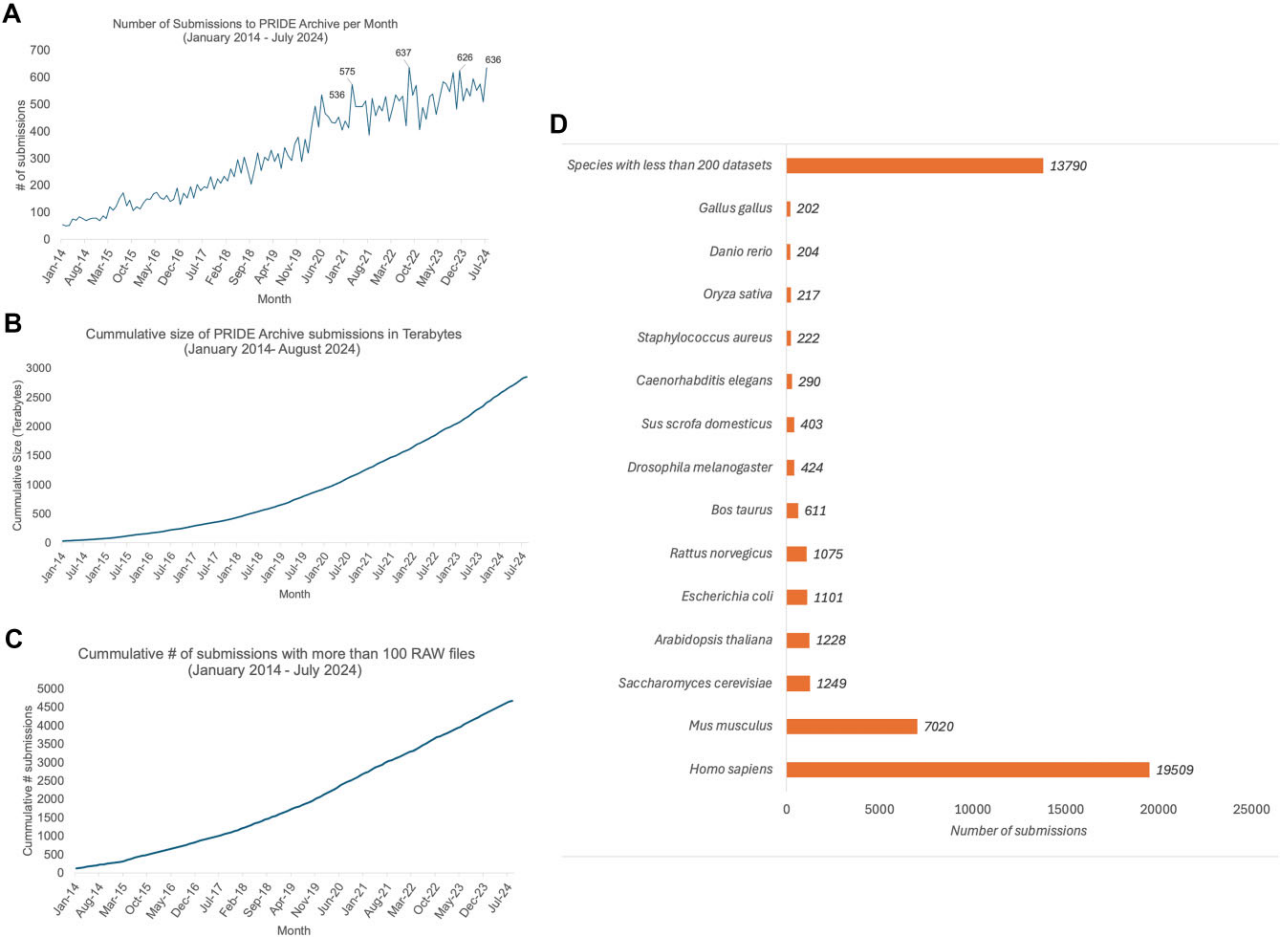
(**A**) Number of submitted datasets to PRIDE Archive per month (from the beginning of ProteomeXchange in 2012 till August 2024). (**B**) Cumulative size of PRIDE Archive data since 2012. (**C**) The number of submitted datasets per species or taxonomy identifier as of August 2024. All species that had <100 datasets are grouped in one category. (**D**) Distribution of the number of submitted datasets to PRIDE Archive per annotated disease.

As of August 2024, PRIDE Archive stored 42 036 datasets—compared to the 23 168 datasets available in August 2021, which means that 44.9% of the datasets in PRIDE Archive have been submitted in the last three years. Figure 3 shows the distribution of submitted datasets per month, species, disease and tissue in PRIDE Archive (January 2014 to August 2024), and the cumulative size of PRIDE Archive in terabytes. In 2023, the average number of submissions was 534 datasets per month. A new highest number of submissions in a single month was achieved in July 2024 (636 datasets) (Figure 3A). Approximately, 69% of the datasets in PRIDE Archive are public (29 039) and 31% private (still unreleased). The percentage of public datasets has steadily increased from 56% in 2019 (42) to 64% in 2021 (3), and now stands at 69%, reflecting our efforts to reduce the time for datasets to remain private.

Two important factors that influence the design of the PRIDE Archive infrastructure are the continued increase in the volume (size) (Figure 3B) of datasets but also in the number of files per dataset. The size of the PRIDE Archive in March 2021 (3) was 1.35 Petabytes. As of August 2024, that size had more than doubled to 285 Petabytes. As a result, PRIDE Archive is the third-largest omics Archive at EMBL-EBI only exceeded by the genomics resources ENA (European Nucleotide Archive) and EGA (European Genome-phenome Archive) (43). More importantly, the average number of files per dataset continues to grow. As of August 2024, >10% of the datasets in PRIDE contained >100 MS raw files (Figure 3C). In December 2023, we processed the largest submission to PRIDE Archive to date (dataset PXD042233), containing 7444 raw files and >15 000 files in total (44).

In terms of taxonomy distribution, as of August 2024, the majority of datasets are from human origin, including those from cell lines (19 509 datasets, 46.4%), followed by mouse datasets (7 020 datasets, 16.7%) and the rest of the main model organisms (Figure 3D). These figures have not changed significantly over the years. The distribution of submitted datasets per disease shows that the majority of datasets are annotated as ‘disease-free (healthy/normal samples)’ followed by datasets generated in studies involving cancer, Alzheimer’s disease and Parkinson’s disease.

## Data reuse activities

Enabling proteomics data reuse, following the FAIR data principles, has been one of the fundamental goals of PRIDE and ProteomeXchange (1,2). Data reuse of PRIDE datasets for multiple applications continuous to increasing. Multiple resources systematically reanalyze datasets from PRIDE including OpenProt (45) (for proteogenomics data), MatrisomeDB (36) (focused on the characterization of enriched extracellular matrix proteins), Scop3P (37) (for PTM data), ProteomeHD (46) (for protein co-expression networks) and PeptideAtlas (7), among others. A recent review from our team (2) shows the overlap in the number of datasets reanalyzed by all these databases. In this context of data reuse, it is also important to highlight the increased importance of PRIDE public datasets in the context of the development of machine learning/deep learning approaches, which are revolutionizing the field (47,48).

**Figure 4.**
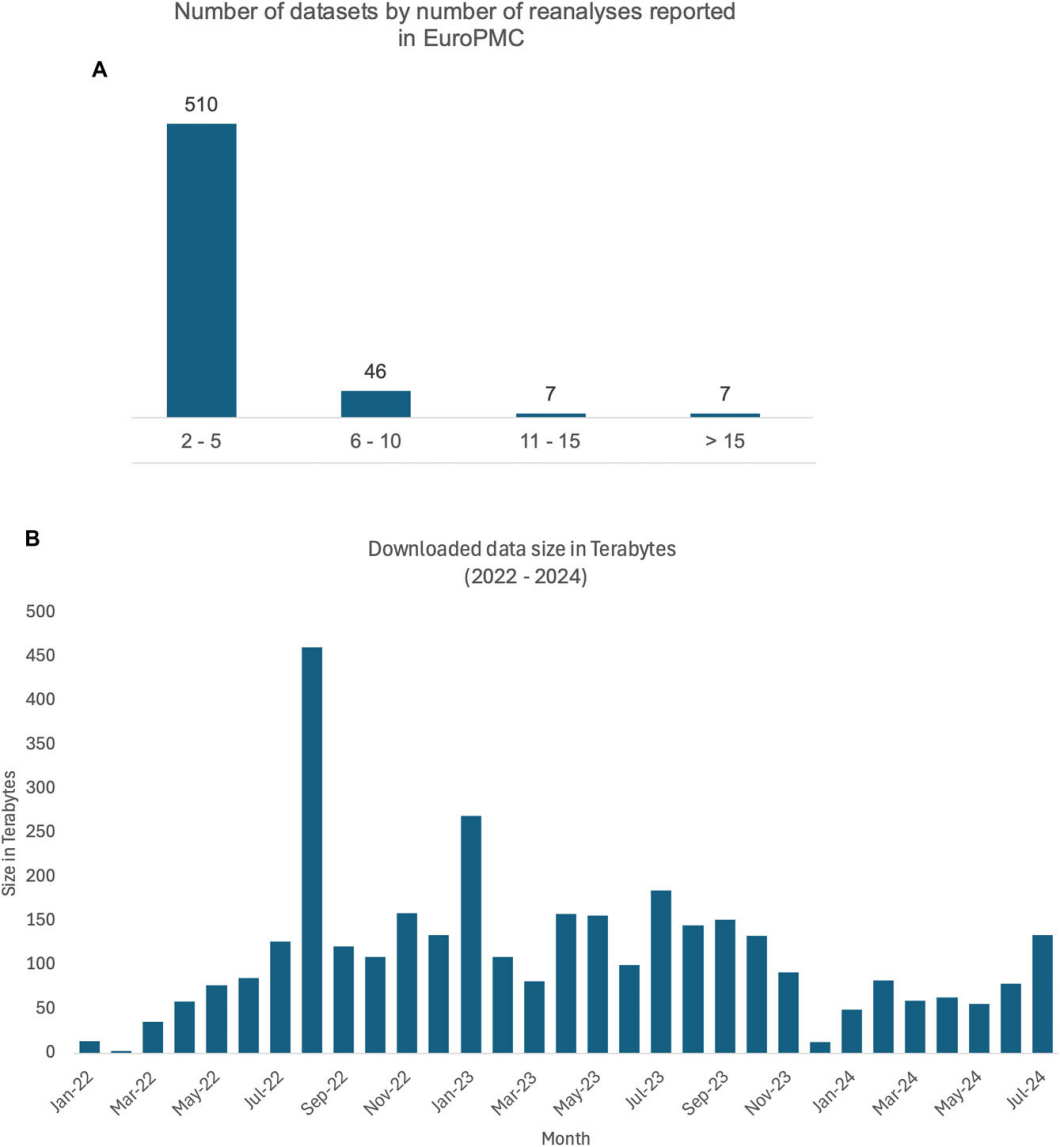
Analysis of PRIDE dataset reuse and data download statistics. (**A**) Distribution of datasets based on the number of reanalyzes reported in EuroPMC. (**B**) Downloaded data size in terabytes from PRIDE Archive per month between 2022 and 2024. The highest volume of data downloads took place September 2022, with over 450 TB. Data download trends fluctuate across months, with other notable peaks observed in January 2023 and July 2024.

Figure 4A shows the number of reanalyzed datasets by counting their direct mentions (dataset accession numbers) in EuropePMC (Europe PubMedCentral) (49). The majority of the datasets are mentioned between 2 and 5 times. However, some of the datasets are reanalyzed multiple times. Overall, the number of datasets mentioned as reanalyzed is <10% of the PRIDE public datasets. The volumes of data downloaded from PRIDE Archive show that on average (from January 2022 to July 2024) >100 TBs were downloaded every month (Figure 4B).

In the context of in-house data reuse, as mentioned above, our focus has been mainly put in disseminating and integrating PRIDE data into added-value EMBL-EBI resources such as UniProt, Ensembl and Expression Atlas. The dissemination of public proteomics data into different resources has different goals depending on each specific resource.

### PRIDE large-scale proteogenomics reanalysis

In 2019, we introduced a mechanism to register ‘TrackHubs’ containing proteomics data in Ensembl, each consisting of a BED file containing peptide coordinates at a genome level paired with the corresponding metadata. We generated ‘TrackHubs’ for over 4 million canonical peptide sequences from 184 PRIDE datasets. Recently, we have developed a new approach that allows PRIDE users to include BED files directly in their submitted datasets. These files can be manually uploaded and visualized into the Ensembl (23) genome browser. To assist PRIDE submitters in converting their peptide identifications into BED files, including genome coordinates, we developed the PepGenome tool (https://github.com/bigbio/pepgenome/), a Java command-line utility. PepGenome converts peptide identification files, such as mzIdentML, mzTab or tab-delimited files, into BED files. The tool supports mapping canonical peptide sequences (peptides with an exact match to the genome/proteome under study) as well as variant peptides, including those with one or more mismatches. Users can include the generated BED files in their data submissions, and after publication, a unique URL will be provided to facilitate loading the data into genome browsers (e.g. https://www.ebi.ac.uk/pride/archive/projects/PXD029362).

We have been recently working on the development of large-scale data workflows for proteogenomics analysis, aiming to map non-canonical peptides, including variants and mutations, to genome coordinates. The quantms workflow (34), an nf-core open-source tool based on OpenMS (50) and DIA-NN (51), enables the reanalysis of public data on cloud and HPC infrastructures using BioContainers packages (52,53). By leveraging custom proteogenomic databases generated with pypgatk (https://github.com/bigbio/py-pgatk) and quantms, we identified 43 501 non-canonical peptides and 786 variant peptide sequences across four public datasets (54). All variant data, along with BED files, were made available in PRIDE Archive (datasets PXD029362 and PXD029360). Additionally, we have recently explored the identification of genome population variants (pangenome) in large-scale tissue proteomes (55), investigating the potential impact of pangenomes on future proteomics experiments and the need for novel workflows to identify and validate non-canonical peptides. We managed to identify 4991 novel peptide sequences and 3921 SAAVs, corresponding to 2367 genes across five population groups.

### PRIDE data dissemination into UniProt

We continue to work under the umbrella of the ‘PTMeXchange’ project (https://www.proteomexchange.org/ptmexchange/, in collaboration with UniProt, PeptideAtlas, Prof. Andy Jones’s team at the University of Liverpool and others), aiming to reanalyze, and disseminate high-quality PTM data from PRIDE and PeptideAtlas into UniProt. First of all, a methodology based on the use of decoy-amino acids was developed to provide a reliable way to calculate the False Localization Rate for phosphorylation (56) and also applied to other PTMs. The data reanalysis work is organized in groups of datasets or ‘builds’, which correspond to the analysis of one particular PTM in one given species. As of August 2024, the builds already finished and integrated in UniProt are phosphorylation in two species: rice (57) and *Plasmodium falciparum* (58). There are other ongoing ‘builds’ at different stages of completion such as human, mouse and *Saccharomyces cerevisiae* phosphorylation, and also work in other PTMs such as human ubiquitination, SUMOyliation and lysine acetylation.

### PRIDE integration of quantitative datasets in Expression Atlas

We have continued to increase the content of reanalyzed quantitative proteomics datasets in Expression Atlas. As of August 2024, Expression Atlas includes protein abundance results coming from 109 proteomics datasets. Most of the integrated datasets come from tissue samples generated in healthy/baseline conditions using DDA approaches. This includes data coming from human samples (32 organs represented) (59), and from model organisms such as mouse (13 organs) and rat (8 organs) (60), and farm pig (14 organs) (61). Additionally, the second main focus comes from datasets generated from cell lines/cancer tissue (62) (the first group of datasets integrated in Expression Atlas), also including a recent study involving the reanalysis of 12 datasets to detect biomarkers of colorectal cancer using public proteomics datasets (63). There is also ongoing work to integrate an additional set of datasets coming from human baseline tissues, but this time generated using DIA approaches (64), following a previous pilot study (65). Data integration between transcriptomics and proteomics datasets in Expression Atlas is enabled because protein abundance is reported in a gene-centric manner.

## Discussion and future plans

Public data deposition and dissemination have revolutionized the proteomics field since the first implementation of the ProteomeXchange data workflow. The proteomics community is widely embracing open data policies. At the same time, public proteomics data are being increasingly reused with multiple applications, with an increasing focus on ‘big data’ approaches. We next outline some of the main working areas for PRIDE in the near future.

PRIDE is enhancing metadata annotation standards for submitted datasets by improving the adoption of the SDRF-Proteomics format, which is increasingly supported by workflows, bioinformatics tools and annotation platforms. SDRF-Proteomics now includes support for use cases such as crosslinking MS and top-down proteomics. Additionally, the proteomics community actively contributes to the annotation of existing PRIDE/ProteomeXchange public datasets within the SDRF-Proteomics file repository (https://github.com/bigbio/proteomics-sample-metadata). Furthermore, we will continue to contribute to other community initiatives such as ProteomicsML (66), aimed at improving data reuse of public datasets for AI approaches.

Additionally, we have recently started to develop a new section of PRIDE Archive for Affinity proteomics (AP) datasets, coming from technologies such as Olink or SomaScan. AP experiments are becoming very popular, especially for human plasma studies, and most datasets are currently not deposited in the public domain, which is a regrettable situation. We are currently working with potential submitters of AP experiments to get the first submissions into the system.

In addition, we have started to work in a controlled-access infrastructure supporting sensitive human proteomics data. This development is needed in the field since there is an increasing number of datasets (including AP datasets) that cannot be made openly available in resources such as PRIDE (or in any other ProteomeXchange resource) due to different legal reasons, including risks related to the identifiability of individuals (67), patient consent agreements and general legislation such as GDPR (Guidelines for Data Protection Regulation) in Europe and HIPPA (Health Insurance Portability and Accountability Act) in the USA (25). We hope a first version of the resource will be available in 2025.

Additionally, we aim to increasingly perform in-house data reuse (including data reanalysis) and disseminate high-quality proteomics data from PRIDE into EMBL-EBI resources. The Open Targets platform (68) will be the next resource where PRIDE data will be integrated, starting with protein quantitative datasets.

The team remains committed to developing tools, workflows, and perform studies that demonstrate how public proteomics data can be reanalyzed to uncover new biological insights. In this context, we have also been working recently in prototype open pipelines for the reanalysis and integration of proteoform-centric data coming from top-down proteomics datasets (69), and data integration between PRIDE with the Human Proteoform Atlas (70) and UniProt in this context remains a possibility for the future.

To finalize, we recommend interested parties in PRIDE-related developments to follow the PRIDE X account (@pride_ebi). For regular announcements of all the new publicly available datasets, users can follow the ProteomeXchange X account (@proteomexchange).

## Data Availability

The PRoteomics IDEntifications (PRIDE) database is freely accessible at https://www.ebi.ac.uk/pride/.
